# DNA Aptamers against the Lup an 1 Food Allergen

**DOI:** 10.1371/journal.pone.0035253

**Published:** 2012-04-17

**Authors:** Pedro Nadal, Alessandro Pinto, Marketa Svobodova, Nuria Canela, Ciara K. O'Sullivan

**Affiliations:** 1 Departament d'Enginyería Química, Universitat Rovira i Virgili, Tarragona, Spain; 2 Servei de Recursos Científics i Tècnics, Universitat Rovira i Virgili, Tarragona, Spain; 3 Institució Catalana de Recerca i Estudis Avançats, Barcelona, Spain; Center for Genomic Regulation, Spain

## Abstract

Using in vitro selection, high affinity DNA aptamers to the food allergen Lup an 1, ß-conglutin, were selected from a pool of DNA, 93 bases in length, containing a randomised sequence of 49 bases. ß-conglutin was purified from lupin flour and chemically crosslinked to carboxylated magnetic beads. Peptide mass fingerprinting was used to confirm the presence of the ß-conglutin. Single stranded DNA was generated from the randomised pool using T7 Gene 6 Exonuclease and was subsequently incubated with the magnetic beads and the captured DNA was released and amplified prior to a further round of Systematic Evolution of Ligands by Exponential Enrichment (SELEX). Evolution was monitored using enzyme linked oligonucleotide assay and surface plasmon resonance. Once a plateau in evolution was reached, the isolated DNA sequences were cloned and sequenced. The consensus motif was identified via alignment of the sequences and the affinities of these sequences for immobilised ß-conglutin were determined using surface plasmon resonance. The selected aptamer was demonstrated to be highly specific, showing no cross-reactivity with other flour ingredients or with other conglutin fractions of lupin. The secondary structures of the selected aptamers were predicted using *m-fold*. Finally, the functionality of the selected aptamers was demonstrated using a competitive assay for the quantitative detection of ß-conglutin. . Future work will focus on structure elucidation and truncation of the selected sequences to generate a smaller aptamer for application to the analysis of the Lup an 1 allergen in foodstuffs.

## Introduction

Lupin is an herbaceous plant of the leguminous family belonging to the genus *Lupinus*. Lupin seeds have been used as human food and animal feed since ancient times and is considered a low-cost protein source. It can be cultivated in cold climates, making it attractive in comparison to other protein rich plants and is an excellent food material with a high nutritional value. Lupin flour and seeds, which are widely available as snacks, are also used in bread, cookies, pastry, pasta, sauces, as well as in beverages as a substitute for milk or soy. However, in response to the increasing number of severe cases of lupin allergies reported during the last decade, in December 2008 lupin was added to the list of substances requiring mandatory advisory labelling on foodstuffs sold in the European Union [1], [2]. The globulin fraction of lupin protein has been associated with its allergenicity and consists of two major globulins called α-conglutin (11S and “legumin-like”), accounting for about 33% of the total protein content in *L. albus*, and β-conglutin (7S and “vicilin-like”), which accounts for another 45%. There are two other globulins, γ- and δ-conglutin, which account for 5 and 12%, respectively, and there have been reports that these fractions are also responsible for allergenicity. Thus, all products containing even trace amounts of lupin must be labelled correctly [3] and the International Union of Immunological Societies (IUIS) allergen nomenclature subcommittee recently designated β-conglutin as the Lup an 1 allergen [4]. Currently available commerial enzyme linked immunosorbent assays exploit polyclonal antibodies that are not specific to ß-conglutin [5] and reports in the literature only detail monoclonal IgG antibodies against α-conglutin and IgM antibodies against ß-conglutin [6], [7], and there are no reports or commercial ELISAs for the specific detection of ß-conglutin. There is thus a need for an analytical tool/method that can specifically detect the Lup an 1 allergen, ß-conglutin.

In the middle of the 19th century Darwin outlined one of the most studied mechanisms of evolution, which he termed natural selection [8], and a century later the first evolutionary experiments with nucleic acids were carried out by Spiegelman using an RNA-dependent RNA replicase for the replication of RNA species [9]–[12]. In the beginning of 1990's an artificial in vitro selection technique for the isolation of specific nucleic acid sequences was reported by three laboratories [13]–[15]. The technique used was termed SELEX, which is an acronym for Systematic Evolution of Ligands by Exponential Enrichment [15], and is a procedure in which a large population of random sequences in a synthetically produced library of nucleic acids is used to select specific aptamers by iterative rounds of systematic binding, competition, selection, amplification, and enrichment. The target-aptamer interaction is normally due to the formation of secondary structures that may act as a scaffold for the target [16].

These selected nucleic acid sequences are called aptamers derived from the Latin *aptus*, “to fit” and the Greek word *meros* “part”, and are artificial ligands [13], with the ability to bind to non-nucleic acid target molecules ranging from large complex molecules such as protein [15], [17]–[19] to simple organic small molecules like ATP [20], [21], dyes [13], [22], amino acids [23], [24] or simple small cations [25], with high affinity and specificity, even being capable of discriminating between enantiomers [26]. There have been reports of aptamers for food safety, capable of the detection for biotoxins such as the mycotoxins ochratoxin A [27] and fumonisin B [28] and endotoxin [29], for a range of antibiotics with detection limits ranging from nanomolar to micromolar, e.g. Kanamycin A [30] and B [31], neomycin [32], tetracycline [33], chloramphenicol [34], as well as for various bacterial pathogens, including *Salmonella typhimurium* [35], *Escherichia coli O157:H7* [36] *Listeria monocytogenes* [37] and *Staphylococcus aureus* [38]. Lysozyme is the only example of a food allergen that aptamers have been selected for and both RNA [39] and DNA [40] aptamers have been selected, with equilibrium dissociation constants of 2.8±0.3 nM and 0.8±2.0 nM [41], as measured by fluorescence anistropy at 25°C.

The objective of this work is the selection of a single stranded DNA aptamer that is specific for the Lup an 1 allergen, ß-conglutin. The ß-conglutin subunit from lupin was first purified and was then chemically crosslinked to magnetic beads. The protein-conjugated magnetic beads were evaluated using peptide mass fingerprinting to ensure the presence of the ß-conglutin on the surface of the beads. A DNA library pool with 10^14^ population variability was amplified using primers where the forward primer was phosphorothioated and single stranded DNA was generated using the T7 Gene 6 Exonuclease yielding 93-mer DNA sequences, which were incubated with the protein-conjugated magnetic beads. Each round of SELEX was monitored using PCR, comparing the amount of DNA liberated from the protein-conjugated beads to that obtained from the unconjugated magnetic beads. Evolution was monitored using enzyme linked oligonucleotide assay (ELONA) and surface plasmon resonance (SPR) and after 15 rounds of SELEX the enriched DNA was cloned, sequenced and consensus motifs identified. The affinity and specificity of these motifs were also evaluated and their secondary structure predicted. Finally the aptamers obtained were applied in a competitive ELONA format for the detection and quantification of the ß-conglutin Lupin allergen.

## Materials and Methods

### Reagents

Phosphate buffered saline (10 mM phosphate, 138 mM NaCl, 2.7 mM KCl, pH 7.4), PBS-tween (10 mM phosphate, 138 mM NaCl, 2.7 mM KCl, pH 7.4, 0.05% v/v Tween 20), 3, 3′, 5, 5′ tetramethyl benzidine (TMB), 1-ethyl-3-(3-dimethylaminopropyl) carbodiimide) (EDC), N-Hydroxysuccinimide (NHS) and all other regents were purchased from Sigma (Barcelona, Spain). Sodium chloride, sodium hydroxide 2 M, hydrochloric acid 6 M, concentrated nitric acid, ampiciline sodium salt, LB medium and agar were purchased from Scharlau Chemie S.A. (Barcelona, Spain). Trypsin was purchased from Roche Molecular Biochemicals, and mass spectra standard calibrators kit from Per Septive Biosystems. Dynabeads^®^ M-270 Carboxylic Acid, TOPO TA Cloning^®^ kit, Tfi DNA Polymerase, 10 bp DNA ladder, One Shot^®^ Top 10 Chemically competent *E. Coli*, Ultrapure X-gal from invitrogen (Invitrogen, Spain). T7 Gene 6 Exonuclease was purchased in USB Corporation (Cleveland, Ohio, USA). Certified™ Low Range Ultra Agarose and Precision Plus Protein™ Standards were purchased on Bio-Rad (Barcelona, Spain). Oligonucleotides (HPLC purified and provided lyophilized) were synthesized by Ella Biotech GmbH (Martinsried, Germany). Oligonucleotides and reagents were used as purchased without further purification. All solutions were prepared in high purity water obtained from a Milli-Q RG system (Barcelona, Spain).

### Preparation of protein-conjugated magnetic beads

Proteins from Lupinus albus seeds were extracted, purified and characterized as previously described [42], obtaining a pure isolate of β-conglutin. β-conglutin was conjugated to Dynabeads® M-270 Carboxylic Acid magnetic beads using carbodiimide coupling. Magnetic beads (100 µl, 2×10^9^ beads/ml) were washed with 25 mM MES, pH 5. After washing, the solution was placed beside a magnet for 4 min and the washing solution removed. The 1-ethyl-3-(3-dimethylaminopropyl) carbodiimide) (EDC) solution (50 µl, 50 mg/ml) and N-Hydroxysuccinimide (NHS) solution (50 µl, 50 mg/ml) were then added and incubated for 30 min at room temperature under shaking conditions. Following incubation, the EDC/NHS solution was removed and the magnetic beads were washed twice with 100 µl of 25 mM MES pH 5, and incubated with the target of interest β-conglutin (100 µl, 2 µg/µl) overnight at room temperature under shaking conditions. After incubation, the mixture of magnetic beads and target was placed on the magnet for 4 min and any unbound ß-conglutin was removed. To block unreacted carboxylic groups on the magnetic bead surface 100 µl of 50 mM ethanolamine in PBS (10 mM phosphate, 138 mM NaCl, 2.7 mM KCl, pH 8.0) was added and incubated at room temperature under shaking conditions for 1 hour. After incubation, the solution was again placed in contact with the magnet for 4 min and the ethanolamine solution was removed by washing three times with 100 µl of PBS-tween pH 8 and the ß-conglutin conjugated magnetic beads were resuspended in 100 µl of PBS (10 mM phosphate, 138 mM NaCl, 2.7 mM KCl, pH 7.4).

### Characterization of protein-conjugated magnetic beads

10 µl of the magnetic beads suspension were washed two times with 50 µl ammonium bicarbonate 25 mM, pH 8 and the supernatant was removed after 2 min of incubation, followed by addition of 10 µl of 20 mM DTT in 50 mM ammonium bicarbonate and incubation for 1 h at 56°C. The supernatant was again removed via magnetic separation, and 10 µL of 50 mM iodoacetamide in 50 mM ammonium bicarbonate added and incubated for 20 min at 21°C, protected from light, followed by supernatant removal. Following the reduction and alkylation steps, the proteins on the magnetic bead surface were digested using trypsin in 25 mM ammonium bicarbonate, in a protein/trypsin ratio (w/w) of 1/50, for 16 hours at 37°C prior to sonication for 10 min at 4°C. One microliter of each sample of extracted peptides was spotted onto a MALDI plate, and when it was almost dry 1 µl of the matrix was added (3 mg/ml α-cyano-4-hydroxycinnamic acid matrix in 50% acetonitrile, 0.1% trifluoroacetic acid). Peptides were selected in the mass range of 750–3500 Da, and acquired in the positive reflector mode. All mass spectra were externally calibrated with the Sequazyme peptide mass standards kit and internally with trypsin autolysis peaks, and processed using Data Explorer Software and MASCOT for matching the spectra profile obtained with the NCBI/UniProtKB/TrEMBL database.

### In vitro Selection: SELEX

The DNA library pool used consisted of diverse 93-mer DNA sequences containing a random region of 49 nucleotides flanked by primer regions as a template SLAu: 5′-agc tga cac agc agg ttg gtg n_49_ca cga gtc gag caa tct cga aat-3′, the forward Primer SLFAuPTO: 5′-a*g *c* t*g *ac aca gca ggt tgg tg-3′, * corresponds to a phosphorothioate and the reverse primer SLRAu: 5′-att tcg aga ttg ctc gac tcg tg-3′. In the first two rounds the DNA library pool (0.5 nmol) was denatured at 95°C for 4 min and then allowed to cool at 4°C for 10 min, after which 5 µl of positive magnetic beads (magnetic beads conjugated with ß-conglutin) were added and the volume was brought to 100 µl with the selection buffer (10 mM phosphate, 138 mM NaCl, 2.7 mM KCl, 1.5 mM MgCl_2_, pH 7.4) and the solution incubated for 30 min at 21°C. The solution containing DNA that did not bind with the magnetic beads was removed, and after 3 washes in 500 µl of binding buffer the DNA bound to the magnetic beads was eluted twice with 50 µl Milli-Q water by denaturation at 95°C for 3 min. After the second round a negative selection step was incorporated where the selected DNA (5 µl) was added to 5 µl of ethanolamine-blocked magnetic beads with no protein attached, and, as before, the volume was brought to 100 µl with the selection buffer (10 mM phosphate, 138 mM NaCl, 2.7 mM KCl, 1.5 mM MgCl_2_, pH 7.4) for incubation at 21°C for 30 min and the unbound DNA was then used as template for the next cycle of SELEX.

### Amplification of selected sequences

100 µl of the PCR mixture contained 10 µl template, 0.5 mM MgCl_2_ 0,1 µM primers, 0.2 mM dNTPs, 5 U *Tfi* DNA polymerase, and buffer for *Tfi* DNA polymerase. After a 5 min incubation at 95°C, 18 cycles pilot PCR were carried out using (i) 95°C, 30 s for denaturation; (ii) 58°C, 30 s for annealing; (iii) 72°C, 30 s for elongation, and finally 5 minutes at 72°C. Single stranded DNA (ssDNA) was generated following PCR by addition of 2.5 U/µl of T7 Gene 6 Exonuclease [43], and after 2 hours incubation at 37°C the reaction was stopped by denaturation of the enzyme by heating at 80°C for 10 min, followed by ethanol precipitation to obtain highly purified ssDNA.

### Monitoring the evolution of SELEX using ELONA and SPR

**Figure 1:**
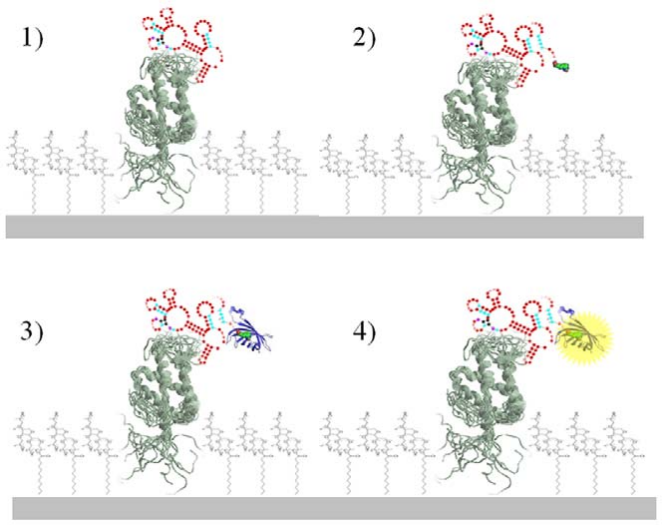
Schematic steps involved in the ELONA type assay. In step 1 the target protein is coated on the surface of a microtitre well and incubated with the aptamer pool. In step 2 biotinylated probe complementary to the constant 3′ primer binding end of the 93-mer DNA is added and hybridises to any DNA that has bound to the immobilised target protein. In step 3, streptavidin labelled-horse radish peroxidase (HRP) is added and binds to the biotin label, and finally in step 4, tetramethylbenzidine substrate for the HRP is added and the resulting increase in absorbance is measured.

ß-conglutin (10 µg/ml) was immobilized on NUNC Maxisorp microtitre plates using 50 mM carbonate buffer pH = 9.6 for 1 h at 37°C, followed by a 1-h blocking with PBS-Tween (10 mM phosphate, 138 mM NaCl, 2.7 mM KCl, pH 7.4, 0.05% v/v Tween 20). The plates were manually washed three times with PBS-Tween. Following these steps aliquots of the same concentration of ssDNA from each of the SELEX cycles in the selection buffer (10 mM phosphate, 138 mM NaCl, 2.7 mM KCl, 1.5 mM MgCl_2_, pH 7.4) were added to each well of the microtitre plate and incubated for 30 min at 21°C. The plates were then manually washed three times with PBS-Tween. 50 µl of 5 nM biotinylated reporter probe (5′-att tcg aga ttg ctc gac tcg tg-3′; 5′-biotinylated) was added to each well and incubated for 1 h at 21°C, again in the selection buffer. The plates were then manually washed three times with PBS-Tween and following the addition of Streptavidin-HRP (0.02 µg/ml) and TMB substrate the reaction was stopped after 20 min with 1 M H_2_SO_4_, and measured at 450 nm using a Spectramax 340PC384 plate reader (Figure 1).

### Cloning and Sequencing

DNA was cloned into the plasmid pCR2.1 using the TOPO TA Cloning^®^ kit (Invitrogen, Spain) according to the manufacturer's instructions. Colonies were subsequently selected and grown overnight in a culture of 5 ml LB medium under vigorous shaking. Plasmid clones were purified with a QIAprep Spin Miniprep kit (Qiagen). Purified plasmid DNA were sequenced by the GenomeLab DTCS Quick Start Kit (Beckman Coulter) according to the manufacturer's instructions, and analyzed in a CEQ8000 Beckman Coulter instrument. The sequences derived were aligned using the Clustal software package of the GCG suite of molecular biology programs and CLC DNA workbench version 5.7.1.

### Surface plasmon resonance (SPR) analysis

BIAcore 3000 (Biacore Inc.) with the Biaevalution software was used for the SPR experiments. Proteins of interest were immobilized, via amine coupling, on separate channels of a CM5 sensor chip. First the chip was activated by EDC/NHS followed by an injection of the protein (5 µl/min for 10 min). After immobilization of the protein any unreacted NHS esters were deactivated by injecting an excess of ethanolamine hydrochloride followed by 75 mM NaOH to remove any non-specific adsorption. The DNA from each cycle of SELEX, as well the final aptamer candidates, were diluted in binding buffer (10 mM phosphate, 138 mM NaCl, 2.7 mM KCl, 1.5 mM MgCl_2_, pH 7.4) and injected for 6 min at a flow rate of 5 µl/min followed by 3 min stabilization and 10 min dissociation. The binding of DNA was analyzed through corresponding changes in the refractive index of optical signals, and expressed as resonance units (RU). All reagents and buffers were prepared in Milli-Q water and were previously filtered.

### Secondary structure prediction

The secondary structure model of the sequences obtained was deduced using m-fold at 21°C in 0.138 M [Na^I^] and 1.5 mM [Mg^II^] folding algorithm and QGRS Mapper, a web-based server for predicting G-quadruplexes in nucleotide sequences [44], [45].

### Competitive ELONA assay

*ß*-conglutin (10 µg/ml) was immobilized on NUNC Maxisorp microtitre plates in 50 mM carbonate buffer, pH 9.6, for 1 h at 37°C, followed by 1 h blocking with PBS-Tween (10 mM phosphate, 138 mM NaCl, 2.7 mM KCl, pH 7.4, 0.05% v/v Tween 20). The plates were then manually washed three times with PBS-Tween. In individual eppendorf tubes, serial dilutions of *ß*-conglutin ranging from 5–100 µg/ml were incubated with Sequence 40 (100 nM) in the selection buffer (10 mM phosphate, 138 mM NaCl, 2.7 mM KCl, 1.5 mM MgCl_2_, pH 7.4) for 30 min at 21°C and then added to the wells of the coated plate and incubated for 30 min at 21°C. The plates were then manually washed three times with PBS-Tween. 50 µl of 5 nM biotinylated probe (biotin-5′-att tcg aga ttg ctc gac tcg tg-3′) was added to each well and incubated for 30 min at 21°C in the selection buffer. The plates were again manually washed three times with PBS-Tween. Following the addition of Streptavidin-HRP (0.02 µg/ml) and after 30-minute incubation, TMB substrate was added, and 15 minutes later 1 M H_2_SO_4_ was added to stop the enzymatic reaction. Finally the absorbance was measured at 450 nm using a Spectramax 340PC^384^ plate reader.

## Results and Discussion

### Evaluation of ß-conglutin conjugated magnetic beads

The attachment of the ß-conglutin to magnetic beads was confirmed using peptide mass fingerprinting (PMF) using the positive controls Prostate Specific Antigen (PSA) and γ-conglutin (purified as described in [42]) with Q8IXI4 and Q9FSH9 accession number in UniProtKB and TreEMBL, respectively (Table 1). Magnetic beads blocked with ethanolamine were used as a negative control. The results obtained from PMF demonstrated clear spectra with a high MOWSE score indicating immobilization of the pure target on the magnetic bead surface. Here the protein attached to the magnetic bead surface was directly digested with trypsin and the peptides produced were then analyzed using peptide mass fingerprinting, and the profile obtained in each spectra was then compared to the NCBI/UniProtKB/TrEMBL databases that contain the theoretical masses derived from the *in silico* tryptic digestion for millions of protein sequences. According to the number of peptide masses matched, including a minimum mass error tolerance of 50 ppm and using the MOWSE score algorithm, the peptide profile in the database were ranked, and the best score identified the target protein, confirming the coupling of the magnetic beads with the ß-conglutin protein [42].

**Table 1:** MOWSE scores obtained in the Peptide mass fingerprinting of the protein-conjugated magnetic beads.

Protein name	MOWSE Score	Protein MW (Da)	pI	Accesion #	Species
β-conglutin (Lup an 1)	4.75E+13	62032	6.1	Q53HY0	*Lupinus Albus*

### In vitro selection: SELEX

The ssDNA library pool was heat-treated to denature any preformed structures and the SELEX procedure started with incubation of the library with the β-conglutin-conjugated magnetic beads in the binding buffer. Following the partitioning of bound from unbound DNA, the selected oligonucleotide pool was amplified using pilot PCR. In the pilot PCR a small aliquot of the SELEX pool was amplified in ranges of 5 to 20 PCR cycles in order to optimise the conditions required for amplification, maintaining the same amount of DNA for each cycle of SELEX, facilitating the use of the same number of molecules in each SELEX round. Once the PCR cycles required to maintain the starting amount of molecules for the next SELEX round was established the selected sequences were then amplified in the final PCR.

Following the amplification of the selected DNA from each SELEX cycle by PCR, double stranded DNA molecules were obtained. The T7 Gene 6 Exonuclease hydrolyzes duplex DNA non-processively in the 5′-3′ direction from both 5′-phosphoryl or 5′-hydroxyl nucleotides by liberating oligonucleotides, as well as mononucleotides, until about 50% of the DNA is acid soluble. To generate single stranded DNA, the forward primer used in the PCR was modified with several phosphorothioates at its 5′end, which protected the forward primer terminated strand of the DNA duplex, liberating ssDNA for the next cycle of SELEX. Following the PCR of each SELEX cycle, the generated ssDNA was directly incubated with the ß-conglutin conjugated magnetic beads as described for the first cycle.

After the second SELEX cycle, prior to the incubation with the protein-conjugated magnetic beads a negative selection step was included, where the amplified DNA, following ssDNA generation was initially incubated with negative magnetic beads (i.e. beads blocked with ethanolamine but without target). This negative selection removes the non-specific sequences that bind to the beads rather than to the target, increasing the stringency of the selection procedure. Pilot PCR was also carried out with oligonucleotides selected with the negative magnetic beads (without target) and with the positive magnetic beads protein-conjugated) for each SELEX cycle and was used to evaluate incremental affinity with each SELEX cycle. After 7 cycles of SELEX a much higher amount of DNA was obtained from the protein-conjugated beads as compared to the non-target conjugated beads, indicating that the DNA pool was becoming more selective towards the ß-conglutin target.

### Affinity and specificity studies

**Figure 2:**
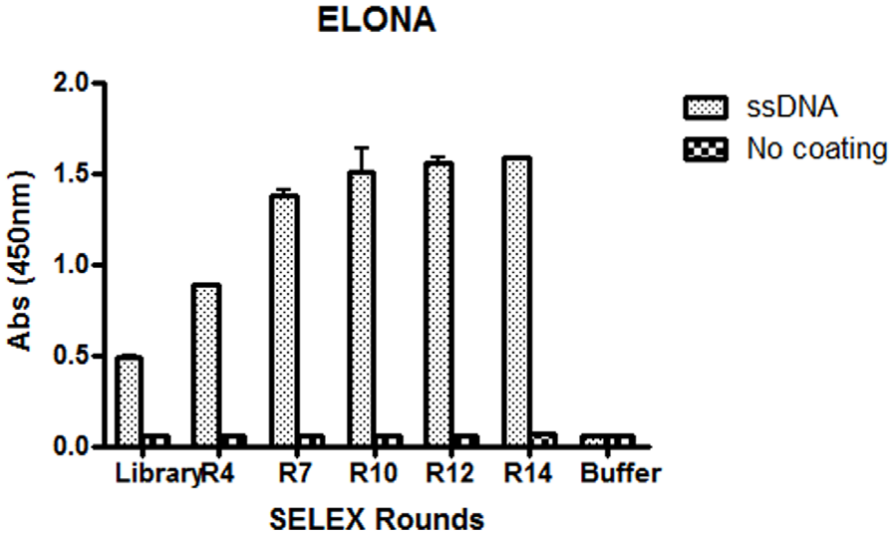
SELEX evolution by ELONA assay. Evaluation of evolution using ELONA monitoring of interaction between immobilised target and DNA isolated after rounds 4 (R4), 7 (R7), 10 (R10), 12 (R12) and 14 (R14), as compared to the interaction with the initial library. A control in the absence of coated target for each of the rounds (No coating) and a background control that refers to the response in the presence of buffer alone (Buffer).

The evolution of the SELEX procedure was tested using enzyme linked oligonucleotide assay (ELONA) and surface plasmon resonance (SPR) using Biacore. For ELONA, in the first step the ß-conglutin target was immobilised on NUNC Maxisorp microtitre plates and then incubated with the ssDNA generated after each cycle of SELEX (Figure 1.1). For the second step a biotinylated probe with the same sequence as the reverse primer, which hybridises to the 3′ end of the selected sequences was added (Figure 1.2), followed by streptavidin-HRP, (Figure 1.3). TMB substrate was then added, providing a colorimetric signal proportional to the amount of aptamer bound (Figure 1.4). This experiment ran the risk that if the reverse primer was involved in the three-dimensional structure of the selected aptamer that no binding of the biotinylated probe would be observed. However, if binding is observed it can be assumed that the primer is not involved in target binding and in truncation studies, could most probably be removed without affecting the affinity of the aptamer. As can be seen in Figure 2, binding was in fact observed, and, in agreement with the PCR results, after the 7th cycle of SELEX, the selected DNA was observed to have increased affinity towards the ß-conglutin target.

**Figure 3:**
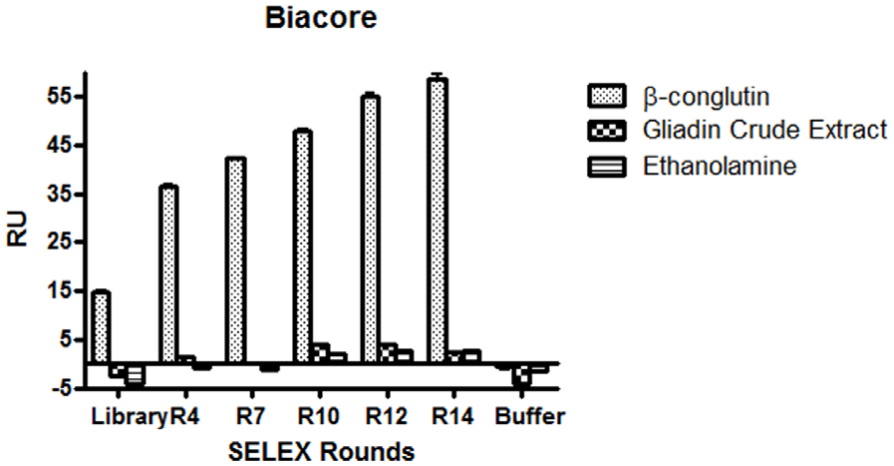
SELEX evolution by SPR. RU reached at the plateau of the association curve for all different channels of the Biacore CM5 chip with DNA isolated from various rounds of SELEX (4, 7, 10, 12 and 14) using channels coated with ß-conglutin, gliadin crude extract and simply blocked with ethanolamine.

The evolution of the DNA pool towards the target protein was also confirmed using surface plasmon resonance with a Biacore3000 and a CM5 chip where the ß-conglutin was attached via amino coupling to the chip surface. In order to check the specificity of the selected DNA, gliadin crude extract, and ethanolamine (blocking agent) were used as negative controls. The selectivity and specificity of the evolving DNA from the SELEX cycles was clearly demonstrated as specific binding was only observed for the β-conglutin channel (Figure 3).

In agreement with the ELONA and PCR results, after the 7th SELEX cycle there is a definitive increase in the affinity of the selected DNA for the target reaching a plateau after the 12–13^th^ cycle (Figure 3). Once evolution had been established, cloning and sequencing was carried out to define the consensus motif.

### Cloning and sequencing

The first step of the cloning was to amplify the SELEX round to be cloned in the same conditions described in the SELEX process. The presence of 3′A-overhangs on the PCR product was incorporated by including a 30 minute extension at 72°C after the last PCR-cycle. These ensure that all PCR products were full length and 3′-adenylated to facilitate ligation of the insert into the plasmid. The lag time extension after the last PCR-cycle favours the nontemplate-dependent terminal transferase activity of the *Tfi* DNA polymerase to add a single deoxyadenosine (A) to the 3′ ends of PCR products and a linearized vector with single, overhanging 3′ deoxythymidine (T) residues allows the PCR inserts to ligate efficiently with the plasmid vector. Following amplification electrophoresis was carried out and the band obtained in the agarose gel was excised and purified for insertion into the plasmid. To ensure that the plasmid contained the aptamer sequence, a PCR was carried out and if the clones contained the aptamer, a 93 bp product should be obtained and thus only clones with a ≈100 bp band were sequenced.

The alignment of the aptamer sequences led to the identification of two sequence families in 50 individual aptamer clones as can be seen in the phylogenetic tree, which describes the relationship between the two groups of sequences obtained (see Figure S1 and S2). This indicates that the initial population of sequences had decreased from 10^14^ different molecules to just a couple of sequence families, indicative of the convergence of potential aptamers in the resulting pool. Consensus motifs were identified from these sequence families and were purchased for analysis of their affinity towards the target protein.

### SPR evaluation of candidate aptamers

The identified consensus motif sequences were evaluated using SPR. Of the 27 sequences listed in Figure S1, which were evaluated for binding to the ß-conglutin target, significant binding was observed for sequences numbered **2** and **40**, detailed here: **S2**: 5′-agc tga cac agc agg ttg gtg ggg gtg gct cac atc atg gta gaa tga ctg aac agc gtt gat taa aag gca cga gtc gag caa tct cga aat-3′ and **S40**: 5′-agc tga cac agc agg ttg gtg ggg gtg gct tcc agt tgg gtt gac aat acg tag gga cac gaa gtc caa cca cga gtc gag caa tct cga aat-3′.

**Figure 4:**
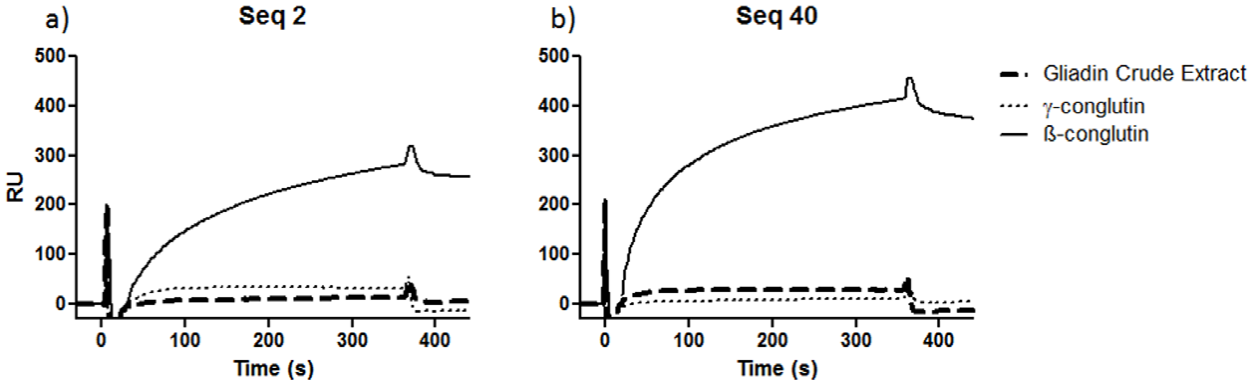
SPR Sensorgrams of the aptamer sequences obtained. a) SPR Sensorgram obtained for Sequence 2 at 10 µM passed through the CM5 Biacore chip surface showing the interaction with the immobilized target (Channel 1: ß-conglutin) and negative controls (Channel 2: γ-conglutin, Channel 3: Gliadin Crude Extract); b) SPR Sensorgram obtained for Sequence 40 at 10 µM passed through the CM5 Biacore chip surface showing the interaction with the immobilized target (Channel 1: ß-conglutin) and negative controls (Channel 2: γ-conglutin, Channel 3: Gliadin Crude Extract).

The signal observed in the negative control channels with immobilised gliadin and γ-conglutin, was negligible for both sequences, demonstrating the high specificity of these aptamer candidates (Figure 4 a) and b)). The K_D_ of each of Sequence **2** and Sequence **40** was obtained by analyzing the binding of a range of concentrations (100 nM to 10 µM) with β-conglutin, using a one to one Langmuir binding model, without mass transfer effect. The resulting K_D_ were 5.15×10^−7^ M, and 3.6×10^−7^ M for sequence **2** and **40**, respectively and a good fit to the model was obtained as demonstrated by the χ^2^ values of 0.0386 and 0.0681 obtained for each sequence (Table 2). [**Note**: The Chi^2^ value is a standard statistical measure of the closeness of fit of data to the model used for elucidation of the K_D_, where for good fitting to ideal data, χ^2^ is of the same order of magnitude as the noise in RU, typically <2].

**Table 2:** Dissociation constants obtained in Biacore with relevant statistics of the aptamers obtained.

Aptamer	Sequence	Model	K_D_ (nM)	X^2^
2	5′-agc tga cac agc agg ttg gtg ggg gtg gct cac atc atg gta gaa tga ctg aac agc gtt gat taa aag gca cga gtc gag caa tct cga aat-3′	Langmuir Binding	515	0.0386
40	5′-agc tga cac agc agg ttg gtg ggg gtg gct tcc agt tgg gtt gac aat acg tag gga cac gaa gtc caa cca cga gtc gag caa tct cga aat-3′	Langmuir Binding	360	0.0681

The values were obtained using duplicates.

The secondary structures of sequence **2** and **40** were modelled using m-fold software [44] and it was predicted that both sequences contain significant secondary structure, including protruding loops and stems (Figure S3). Furthermore, a QGRS-mapper was used to predict putative G-quadruplexes formed from G-Rich Sequences in sequence **2** and **40** (Figure S4), revealing a high probability of the presence of G-quadruplex structures in both sequences. Detailed ongoing studies involving NMR and circular dichroism analysis will provide a more exact description of the aptamer structure, but the QGRS-mapper clearly indicates that G-quartets are involved in the aptamer structure, a property that can be exploited when engineering a molecular beacon structure.

### Competitive ELONA assay

**Figure 5:**
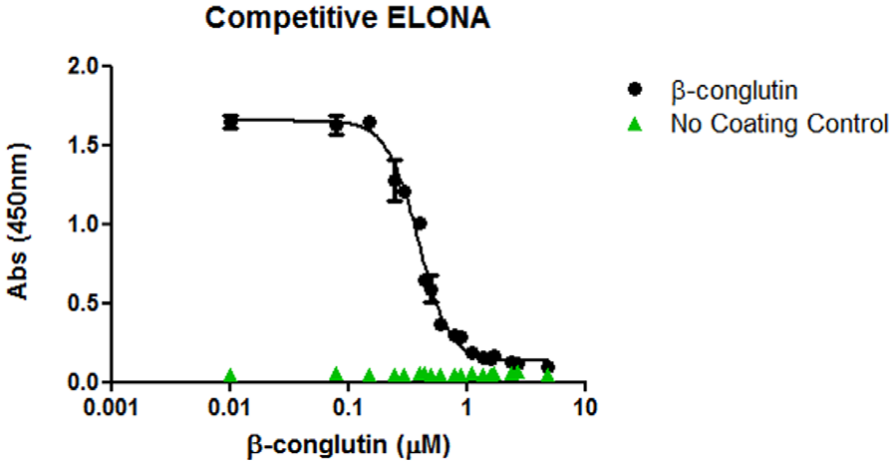
ELONA assay. Detection of ß-conglutin by ELONA using Sequence 40 in SELEX Binding Buffer at 100 nM final concentration.

In order to demonstrate the functionality of the selected aptamer, a competitive ELONA assay was developed for the quantitative detection of ß-conglutin. This assay was based on the use of ß-conglutin immobilised on a microtitre plate, which then “competed” with ß-conglutin analyte for binding to the selected aptamer (sequence 40), where, similiar to competitive ELISA formats, in the presence of higher concentrations of the ß-conglutin analyte, less aptamer bound to the immobilised ß-conglutin, resulting in a lower signal. A calibration curve between 0 and 4.8 µM of ß-conglutin (Figure 5), was obtained, with an EC50 value of 392.8 nM. This EC50, which is the half concentration of a ligand where the response (or binding) is maximal, is in agreement with the K_D_ values obtained using surface plasmon resonance (Table 2). The LOD obtained was 153 nM, and the r^2^ was 0.999, clearly demonstrating the functionality of the selected aptamer for the quantitative detection of ß-conglutin, the identified Lup an 1 allergen.

In conclusion, we report on the use of a SELEX procedure based on the use of protein-conjugated magnetic beads for the generation of aptamers able to bind specifically to the lupin β-conglutin allergen (Lup an 1). The final aptamers obtained detect the allergen Lup an 1 with high affinity and specificity capable of distinguishing it from other possible proteins present in flour, e.g. gliadin, or other globulin proteins present in lupin such as γ-conglutin. Furthermore the secondary structures of both sequences were predicted and evaluated using the GQRS-Mapper obtaining high G-score value for both sequences, indicative of the presence of G-quadruplexes. Finally the applicability of the aptamers obtained had been demonstrated by approaching a competitive ELONA assay. Further work will involve the elucidation of the exact structure of the aptamer and its truncated forms and other applications of those aptamers for the analysis of the Lup an 1 allergen, ß-conglutin, in foodstuffs.

## Supporting Information

Figure S1 **Alignment of cloned sequences in clustalW.**
(TIF)

Figure S2 **Filogenetic tree showing the relationship of the two groups of sequences sequenced in the cloning step.**
(TIF)

Figure S3 **Secondary structure prediction using m-fold software. Sequence 2 on the left, and Sequence 40 on the right.**
(TIF)

Figure S4 **Prediction of Guanine Tetrades.** G-Score Graph for G-Quadruplex structure prediction using QGRS-mapper software, which indicates the probability of finding a G-rich motif capable of forming a G-quadruplex structure. Sequence 2 is shown on the left and Sequence 40 on the right.
(TIF)
