# Supplementary figures and images for: DNA Aptamers against the Lup an 1 Food Allergen
Source: PLoS One. 2012 Apr 17;7(4):e35253. doi: 10.1371/journal.pone.0035253 (PMC3328447; doi:10.1371/journal.pone.0035253)

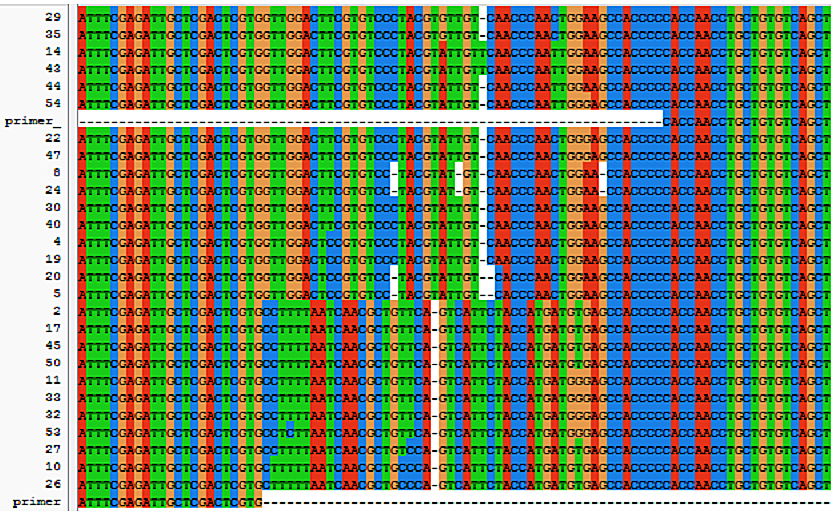

Supplement: Figure S1 — Alignment of cloned sequences in clustalW. (TIF) [file pone.0035253.s001.tif]

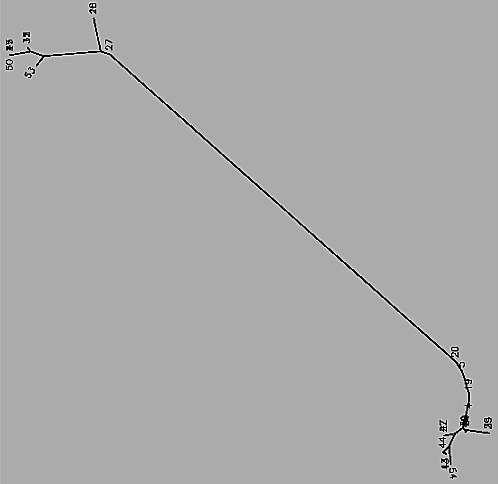

Supplement: Figure S2 — Filogenetic tree showing the relationship of the two groups of sequences sequenced in the cloning step. (TIF) [file pone.0035253.s002.tif]

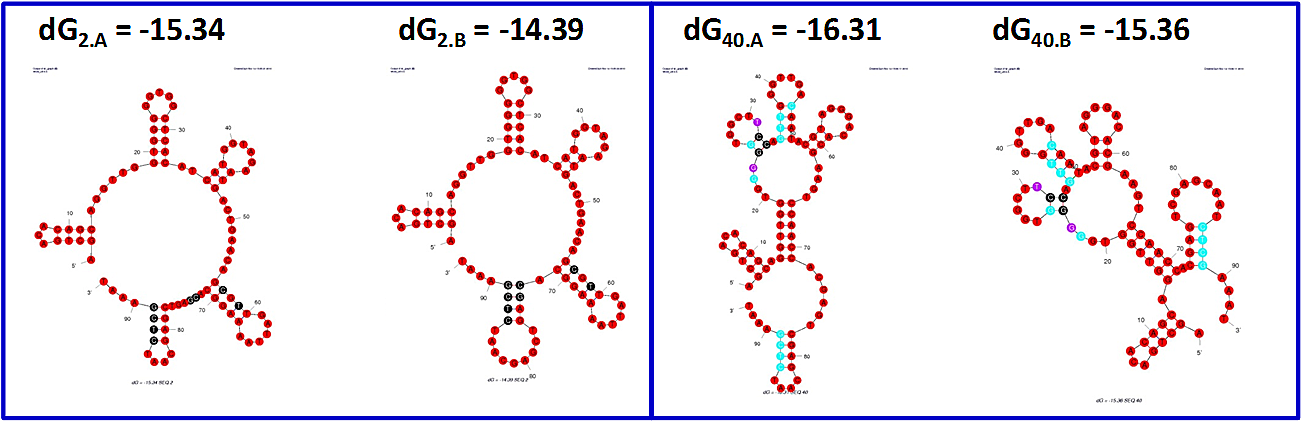

Supplement: Figure S3 — Secondary structure prediction using m-fold software. Sequence 2 on the left, and Sequence 40 on the right. (TIF) [file pone.0035253.s003.tif]

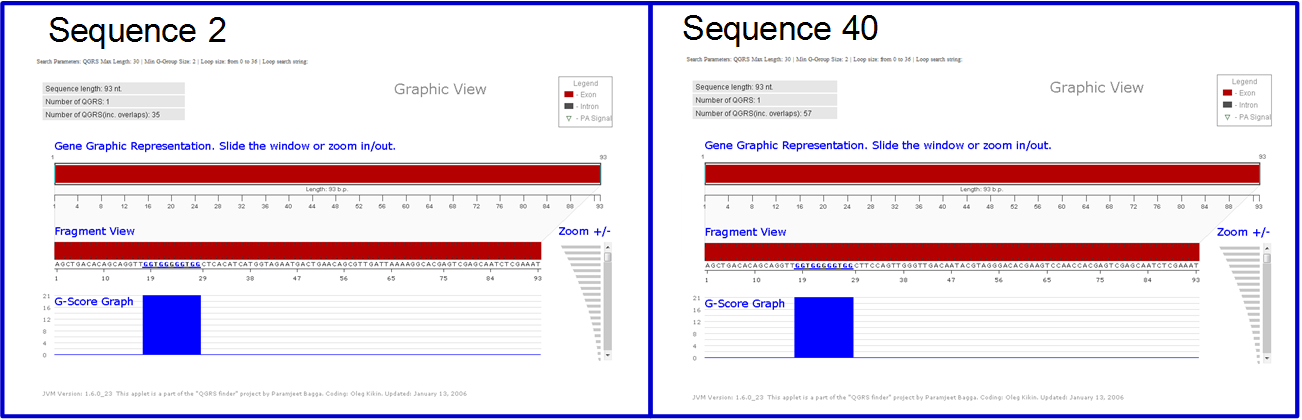

Supplement: Figure S4 — Prediction of Guanine Tetrades. G-Score Graph for G-Quadruplex structure prediction using QGRS-mapper software, which indicates the probability of finding a G-rich motif capable of forming a G-quadruplex structure. Sequence 2 is shown on the left and Sequence 40 on the right. (TIF) [file pone.0035253.s004.tif]
